# An update on the diagnosis and treatment of seborrheic keratosis

**DOI:** 10.3389/fmed.2026.1764657

**Published:** 2026-09-04

**Authors:** Sri Nauli Dewi Lubis, Imam Budi Putra

**Affiliations:** Department of Dermatology and Venereology, Faculty of Medicine, Universitas Sumatera Utara, Medan, Indonesia

**Keywords:** laser treatment, Nano-Pulse Stimulation (NPS), non-invasive diagnosis, seborrheic keratosis, topical therapy

## Abstract

**Background:**

Seborrheic keratosis (SK) is a common benign epidermal neoplasm, particularly affecting the elderly. Although non-malignant, it often poses cosmetic and diagnostic challenges due to its resemblance to malignant skin tumors. This review aims to provide a comprehensive update on the recent advancements in the epidemiology, pathogenesis, diagnostic modalities, and therapeutic approaches for SK.

**Methods:**

A narrative review of current literature was conducted, focusing on molecular insights, diagnostic innovations, and therapeutic options for SK. Sources were identified through searches in PubMed, ScienceDirect, and Google Scholar over the last decade using relevant keywords.

**Result:**

Molecular research has highlighted the role of oncogenic mutations, including AKT pathway alterations and amyloid precursor protein (APP) involvement. Non-invasive diagnostic tools such as dermatoscopy, reflectance confocal microscopy (RCM), and optical coherence tomography (OCT) have improved lesion differentiation. Artificial intelligence (AI)-based algorithms have further enhanced diagnostic precision. While conventional therapies such as cryotherapy, excision, electrodesiccation, and laser remain effective, they may cause post-inflammatory hypopigmentation. Recent non-invasive treatments, including 30%−50% aqueous nitric-zinc oxide solution with organic acids, 65 or 80% trichloroacetic acid (TCA) solution, and 0.3% Annona muricata L. seed extract cream have demonstrated efficacy with minimal side effects. Nano-Pulse Stimulation (NPS) technology emerges as a novel low-complication treatment.

**Conclusion:**

Advances in molecular understanding and technology have transformed the diagnostic and therapeutic landscape for SK. There is a growing need for accessible, effective, and cosmetically favorable treatment options, especially with rising patient demand. Further research is warranted to validate long-term efficacy and broaden availability.

## 1 Introduction

Seborrheic keratosis is a common skin condition, typically presenting as a benign skin tumor with significant clinical variability. Although aging is a known risk factor, the precise roles of ultraviolet (UV) exposure and immune system dysfunction remain unclear. Interestingly, this benign disorder is paradoxically driven by oncogenic mutations, which may have important implications for understanding malignancy. Advances in molecular pathogenesis suggest that inhibition of the AKT pathway and APP, as well as the use of certain existing skin cancer therapies, may hold therapeutic potential for SK. Dermatoscopy criteria have become increasingly important for accurate detection, while other non-invasive diagnostic methods, such as RCM and OCT are rapidly evolving. Given its ability to mimic malignant tumors, SK is frequently utilized in training AI-based algorithms for computerized skin disease detection. These technologies are becoming increasingly accurate and may significantly enhance clinical practice (1, 2).

Current treatment options for SK can cause discomfort and post-treatment side effects, particularly in individuals with darker skin tones. Following the discontinuation of 40% hydrogen peroxide topical solution (ESKATA^®^) at the end of 2019, promising alternatives such as topical 30%−50% aqueous nitric-zinc oxide solution with organic acids (acetic, lactic, and oxalic acids) and 65 or 80% TCA solution/chemical peel require further development. There is also a need for larger and more focused clinical trials of laser therapies to ensure that future treatment standards can meet the diverse needs of patients (2).

Despite being one of the most common benign epidermal tumors, SK continues to present important diagnostic and therapeutic challenges in routine clinical practice. Clinically, SK may mimic malignant and premalignant lesions, creating diagnostic uncertainty and often prompting biopsy or unnecessary destructive treatment. At the same time, the diagnostic landscape is evolving rapidly with the increasing use of dermatoscopy, RCM, OCT, and AI-assisted image analysis. Recent studies have further highlighted the growing role of AI-based dermatologic screening for SK and other common epithelial lesions, as well as the potential of hyperspectral imaging combined with machine learning to improve skin lesion classification. In parallel, although conventional therapies such as cryotherapy, curettage, electrodessication, and laser remain widely used, they may be limited by pain, pigmentary alteration, scarring, recurrence, and suboptimal cosmetic outcomes, particularly in patients with multiple lesions or darker skin types (1, 2). Therefore, this review aims to provide an updated and clinically oriented synthesis of SK, with emphasis on current gaps in diagnosis, recent technological advances, and evolving treatment paradigms, in order to support more accurate diagnosis and more effective, cosmetically acceptable management.

## 2 Methods

This article was conducted as a structured narrative review to summarize recent advances in the diagnosis and treatment of SK. A literature search was performed in PubMed, ScienceDirect, and Google Scholar for articles published from January 2017 to December 2025 using combinations of the following terms: “seborrheic keratosis,” “diagnosis,” “non-invasive techniques,” “dermatoscopy,” “dermatoscopy,” “reflectance confocal microscopy,” “optical coherence tomography,” “artificial intelligence,” “hyperspectral imaging,” “topical therapy,” “laser treatment,” “nitric-zinc,” “trichloroacetic acid,” “hydrogen peroxide,” “Nano-Pulse Stimulation,” and “Annona muricata.”

Eligible articles included original research, clinical trials, observational studies, systematic reviews, narrative reviews, and well-documented case reports that provided relevant insights into the epidemiology, pathogenesis, diagnosis, or treatment of SK. Articles were excluded if they were duplicate records, not written in English, lacked sufficient methodological detail, or were not directly relevant to the clinical focus of this review. The initial search identified records from PubMed, ScienceDirect, and Google Scholar. After screening for relevance and eligibility, 19 studies were included in the qualitative synthesis.

Because this study was designed as a narrative review rather than a systematic review, formal meta-analysis was not performed. However, the included literature was critically appraised and categorized according to study design and level of evidence, including randomized or controlled trials, observational studies, pilot studies, case series, case reports, and experimental or preclinical studies. Particular attention was given to reported efficacy, recurrence, adverse effects, cosmetic outcomes, and methodological limitations of newer treatment modalities.

## 3 Result

To improve methodological transparency, the main studies included in this review are summarized according to study design, focus, principal findings, and limitations (Table 1).

**Table 1:** Summary of included studies on diagnostic and therapeutic advances in SK.

References	Study design	Focus/topic	Main findings	Key limitations	Evidence category
Sun and Halpern, (2)	Narrative review	Etiology, diagnosis, and management update	Summarized recent advances in SK biology, diagnosis, non-invasive imaging, AI, and treatment options	Narrative design without formal systematic selection	Review
Minagawa, (13)	Observational/dermatoscopic study	Dermatoscopy–pathology correlation in SK	Demonstrated characteristic dermatoscopic findings useful for differentiating SK from melanocytic lesions	Observational design and limited histopathologic correlation	Observational
Weber et al. (14)	Review of diagnostic imaging	Dermatoscopy of neoplastic skin lesions	Confirmed diagnostic utility of dermatoscopic criteria in routine clinical differentiation of SK	Interpretation remains operator-dependent	Observational/diagnostic review
Khalili et al. (12)	Clinicopathological study	Clinical and histopathologic subtypes of SK	Demonstrated heterogeneity of SK morphology and histopathological variants	Single-center design and possible spectrum bias	Observational
Guo et al. (15)	Retrospective imaging study	RCM in SK	RCM improved lesion characterization but had limitations in distinguishing atypical lesions	Limited penetration depth and diagnostic variability	Observational
Marchetti et al. (16)	Algorithm validation study	AI in lesion classification	AI algorithms showed promising accuracy in distinguishing SK from melanoma	Dependent on training datasets and external validation	Diagnostic technology
Lin et al. (24)	Clinical AI-assisted screening study	AI-assisted dermatologic screening	Highlighted growing utility of AI for screening epithelial skin lesions including SK	Performance may vary across datasets and clinical settings	Diagnostic technology
Lin et al. (25)	Imaging/machine learning study	Hyperspectral imaging and machine learning	Suggested improved lesion classification beyond conventional imaging	Early-stage evidence requiring broader validation	Diagnostic technology
Keaney, (19)	Controlled clinical review	Hydrogen peroxide 40% w/w topical solution	Demonstrated lesion clearance in a proportion of treated SK lesions	Complete clearance not universal and later commercial discontinuation	Controlled trial
Barthelmann et al. (17)	Review/clinical update	30–50% aqueous nitric-zinc oxide solution with organic acids and emerging topical therapies	Reported encouraging clearance and tolerability of 30–50% aqueous nitric-zinc oxide solution with organic acids therapy	Small studies and short follow-up	Early clinical evidence
Komalasari et al. (7)	Case report	TCA therapy	Showed favorable response with topical TCA in atypical SK lesion	Limited to case-based evidence	Pilot/early clinical evidence
Elvira et al. (21)	Clinical study	0.3% Annona muricata L. seed extract cream	Reported clinical improvement using topical soursop seed extract	Small sample size and limited external validation	Pilot/early clinical evidence
Zaresharifi et al. (18)	Comparative clinical study	Laser therapy (CO_2_, Er:YAG)	Demonstrated efficacy with variable cosmetic outcomes and pigmentary effects	Limited long-term recurrence data	Clinical procedural evidence
Barthelmann et al. (17)	Clinical review	Nano-Pulse Stimulation (NPS)	Reported promising lesion clearance and patient satisfaction	Limited comparative studies and short follow-up	Emerging procedural evidence

### 3.1 Definition

SK is a commonly encountered skin condition and one of the most prevalent benign epidermal tumors. Although it is benign, removal may be considered for cosmetic or diagnostic reasons, particularly in cases of the irritated type or melanoacanthoma variant. The face and upper trunk are the most common sites of predilection, although lesions can occur on any part of the body (3).

### 3.2 Epidemiology

SK is highly prevalent among the elderly population and appears to increase with age. In a recent retrospective cohort study of 10,545 Brazilian patients aged over 60 years, SK was found to be the leading cause of dermatologic teleconsultation. Notably, 89% of lesions were reported in patients aged 80 years and older. Previous studies conducted in Australia, the United Kingdom, the Netherlands, and South Korea similarly reported high prevalence rates strongly associated with age, along with higher average numbers and diameters of lesions among geriatric individuals. However, SK can also develop in younger individuals. Another Australian study involving 170 people aged 15–30 found that 24% had at least one lesion, with 78% of these located on the trunk. Although pediatric dermatologists rarely encounter SK, the dermatosis papulosa nigra (DPN) variant has been specifically observed in children (4).

SK is commonly noted as an incidental finding in epidemiological studies, and relatively little is known about its demographic distribution. Recent research has found no significant gender differences in SK incidence, although observational studies suggest that men tend to have more lesions on the trunk and arms than women. While previously assumed to be more common in Caucasians, SK appears to occur with similar frequency in Korean men (Fitzpatrick skin types I–V) and is now recognized across a variety of skin types. DPN is more frequently observed in African-American, East Asian, and South Asian populations (Fitzpatrick types IV–VI), with consistently reported prevalence among women, though it remains underreported. A strong familial history is often noted, especially in individuals with numerous lesions. An autosomal dominant inheritance pattern with incomplete penetrance has been proposed (5).

### 3.3 Risk factors

UV exposure has been implicated in the etiopathogenesis of SK, although a direct causal relationship remains unclear. A study conducted in Australia found that SK lesions were more frequent, flatter, and larger in sun-exposed areas of the body, and that the overall prevalence of SK was higher compared to other Caucasian populations in the United Kingdom. Similarly, the aforementioned study involving Korean men found that lesions were concentrated on the face and dorsal hands, with a 2.28-fold increased risk of SK among individuals with more than six hours of daily lifetime sun exposure compared to those with less than three hours per day. A survey of 840 individuals in China also identified a correlation between cumulative UV exposure and SK, noting that individuals with Fitzpatrick skin type II were more susceptible to severe photoaging compared to those with types III and IV. However, a cohort study of 966 individuals in the Netherlands found no significant association between lifetime sun exposure or painful sunburns and increased SK risk. Interestingly, SK lesions frequently occur in areas typically covered by clothing and have been observed in perigenital and intertriginous regions. Notably, SK is more commonly associated with atrophic rather than hypertrophic photoaging (6).

Age is a widely recognized risk factor for SK. The APP, which plays a significant role in the pathogenesis of age-related Alzheimer's disease, has recently been shown to be upregulated in SK tissue compared to controls. Its expression levels were significantly higher in UV-exposed skin and among older age groups (61–85 years), although age-related positive correlations were observed in the epidermis but not the dermis. Partial loss of presenilin 1 and 2—enzymes involved in the release of beta-amyloid from APP in neurological disorders—has been shown to cause SK-like epidermal hyperplasia in mouse models. Furthermore, the upregulation of guanine deaminase (GDA) in SK has also been implicated in the condition's pathogenesis and pigmentation (26, 27).

### 3.4 Classification

#### 3.4.1 Clinical variants

##### 3.4.1.1 Common seborrheace keratosis (CSK)

**Figure 1:**
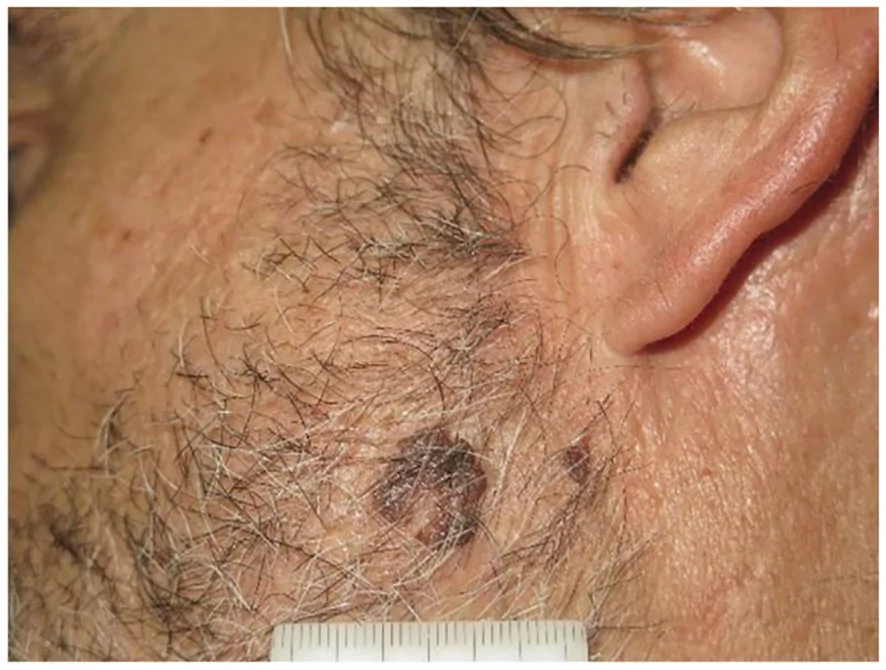
Clinical presentation of seborrheic keratosis. Reproduced from Komalasari et al. (7) under the creative commons attribution 4.0 international license (CC BY 4.0).

Clinically, CSK presents as well-demarcated, brown papules with a “stuck-on” appearance and a velvety or granular surface. It typically occurs on hair-bearing areas of the body. The lesions may vary in color, including black, white, yellow, maroon, and grayish-blue. CSK is slightly more frequent in men. Initially, the lesions may appear flat but tend to become verrucous as they grow. Histopathologically, CSK is characterized by dominant orthokeratotic hyperkeratosis with papillomatosis. True horn cysts are rare or absent, while pseudo-horn cysts are commonly observed (7). A representative clinical presentation of seborrheic keratosis is shown in Figure 1.

##### 3.4.1.2 Dermatosis papulosa nigra (DPN)

DPN primarily affects individuals with darker skin types and clinically appears as hyperpigmented, round or filiform papules with a smooth surface, measuring approximately 1 to 5 mm. It is more commonly observed in women and typically affects the face and upper trunk. A positive family history is frequently noted (8).

##### 3.4.1.3 Stucco keratosis

These lesions affect the lower legs, especially around the ankles, and are more commonly seen in elderly men. Clinically, they appear as wart-like papules with a white to yellow coloration and a characteristic “stuck-on” appearance. The lesions are easily detachable and may bleed slightly when scraped (2).

##### 3.4.1.4 Inverted follicular keratosis (IFK)

In middle-aged and elderly individuals, IFK often presents as asymptomatic, isolated, whitish to pinkish lesions on the face, typically the cheeks and upper lip. Cases have also been reported on the conjunctiva. Multiple IFKs may occur in Cowden syndrome, serving as cutaneous markers. Due to its resemblance to squamous cell carcinoma (SCC), a diagnostic biopsy with histological examination is recommended when the diagnosis is uncertain (9).

##### 3.4.1.5 Lichenoid keratosis (LK)

LK clinically presents as slightly elevated reddish-brown plaques, often located on the upper chest, face, or forearms. It is more frequently seen in elderly Caucasian women, typically between the fourth and seventh decades of life. Non-scaling lesions may resemble basal cell carcinoma (BCC), making LK diagnostically challenging (10).

##### 3.4.1.6 Flat seborrheic keratosis

**Figure 2:**
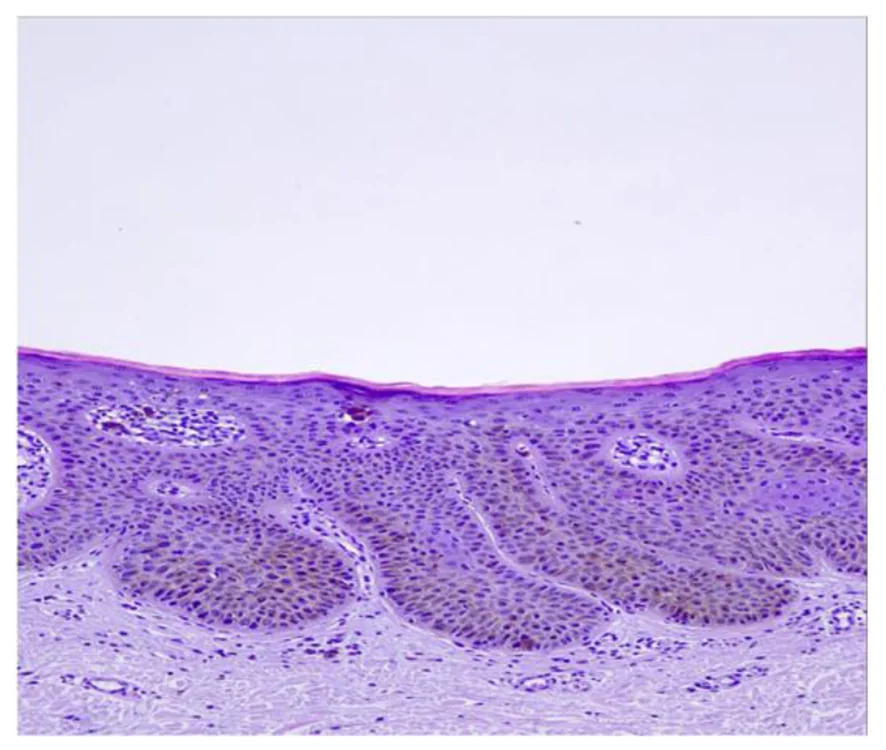
Flat/macular seborrheic keratosis. Reproduced from Khalili et al. (12) under the creative commons attribution license (CC BY 4.0).

These lesions appear as brownish macules in sun-exposed areas of the body and closely resemble solar lentigines. They tend to enlarge progressively with age and are usually asymptomatic (11). A representative histopathological image of flat or macular seborrheic keratosis is shown in Figure 2.

##### 3.4.1.7 Large cell acanthoma (LCA)

On sun-damaged skin, LCA manifests as solitary, scaly, reddish-brown or orange papules or plaques, often described as “stuck-on” lesions. They most commonly affect the neck and face, including the eyelids, but may also involve the trunk and extremities. Hypopigmented or skin-colored variants are not uncommon (12).

#### 3.4.2 Histopatologic variants

##### 3.4.2.1 Hyperkeratotic SK

This variant is characterized by prominent orthohyperkeratosis and papillomatosis. Acanthosis is either mild or absent, and horn cysts are usually not present. Some studies have reported structures resembling “pseudo-horn cysts” when observed in cross-sectional views (12).

##### 3.4.2.2 Retikulat/adenoid variants

This form exhibits a double row of thin reticulated acanthosis along with mild to moderate papillomatosis and hyperkeratosis. Pigmented variants are commonly observed. Horn cysts are rarely found. This subtype is more frequently seen in sun-exposed areas (12).

##### 3.4.2.3 Clonal type

This variant shows pronounced orthohyperkeratosis, acanthosis, and papillomatosis. Tumor cells appear spindle-shaped, and nests or islands of basaloid cells may be present in some lesions (12).

##### 3.4.2.4 Irritated type

Characterized by the proliferation of eosinophilic spindle-shaped tumor cells, sometimes forming circular structures. Dyskeratotic cells may also be present. Differentiating between irritated SK and SCC can be challenging. Dermatoscopy is a helpful tool for this distinction; however, in cases of diagnostic uncertainty, excision followed by histological and immunohistochemical examination is strongly recommended. A recent study highlighted that combining nucleolar small ribonucleoprotein IMP3 U3 with B-cell lymphoma 2 (Bcl-2) regulatory protein provides a protective diagnostic marker (12).

##### 3.4.2.5 Melanoacanthoma

**Figure 3:**
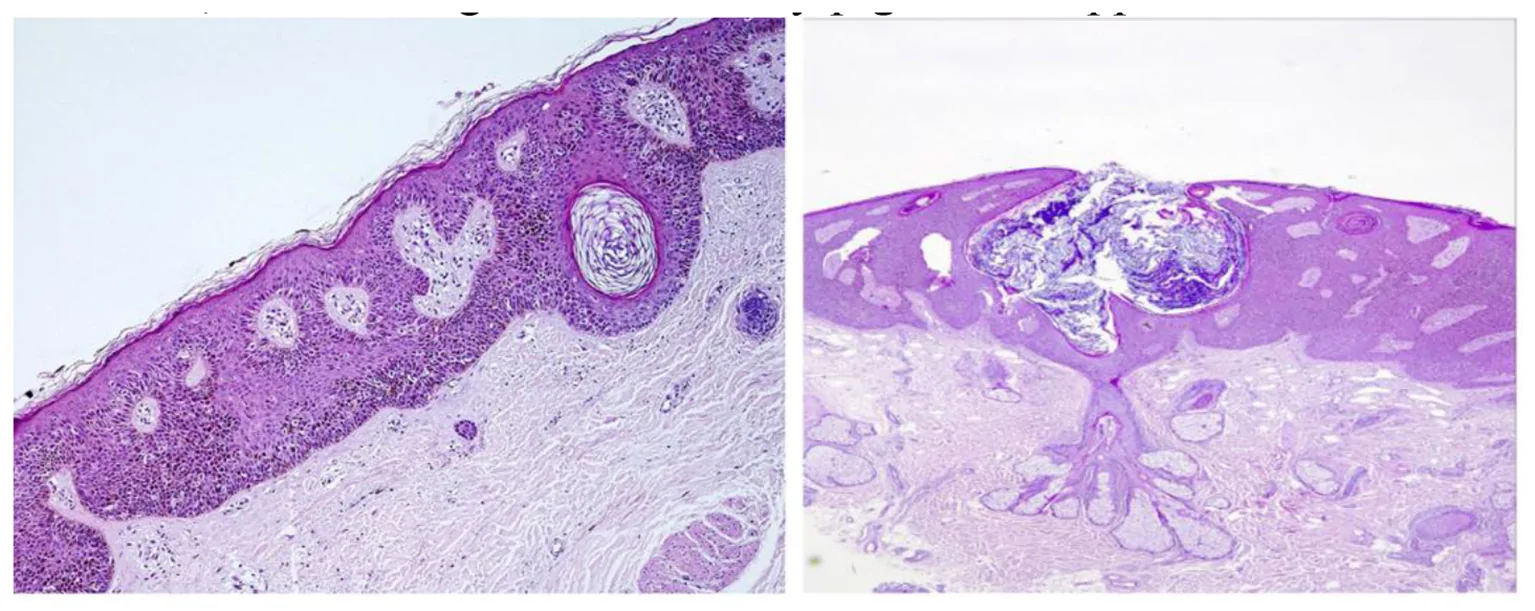
Melanoacanthoma. Reproduced from Khalili et al. (12) under the creative commons attribution license (CC BY 4.0).

This is a pigmented acanthotic variant of SK with minimal or absent hyperkeratosis. It features marked basaloid cell proliferation and a high number of melanocytes. Melanophages may also be present in the dermis, contributing to the intensely pigmented appearance of the lesion (12). Representative histopathological features of melanoacanthoma are shown in Figure 3.

##### 3.4.2.6 Mix adamatanoid type

Histologically, this variant consists of small basaloid keratinocytes that are clearly separated from the surrounding normal epidermis. Multiple pseudo-horn cysts are found throughout the acanthotic epithelium. A distinctive feature of the intraepidermal nests is the presence of granular blue-gray intercellular material, giving the neoplastic cells a pale appearance. Due to mucinous intercellular deposition, the elongated, star-shaped neoplastic cells form delicate bridges across the empty areas (12).

##### 3.4.2.7 SK with pseudo-rosette formation

This acanthotic variant of SK features thickened epidermis with orthohyperkeratosis and pseudo-horn cysts. A key histological feature is the radial arrangement of basaloid keratinocytes around small central spaces, creating rosette-like structures (12).

##### 3.4.2.8 Acanthotic variants

**Figure 4:**
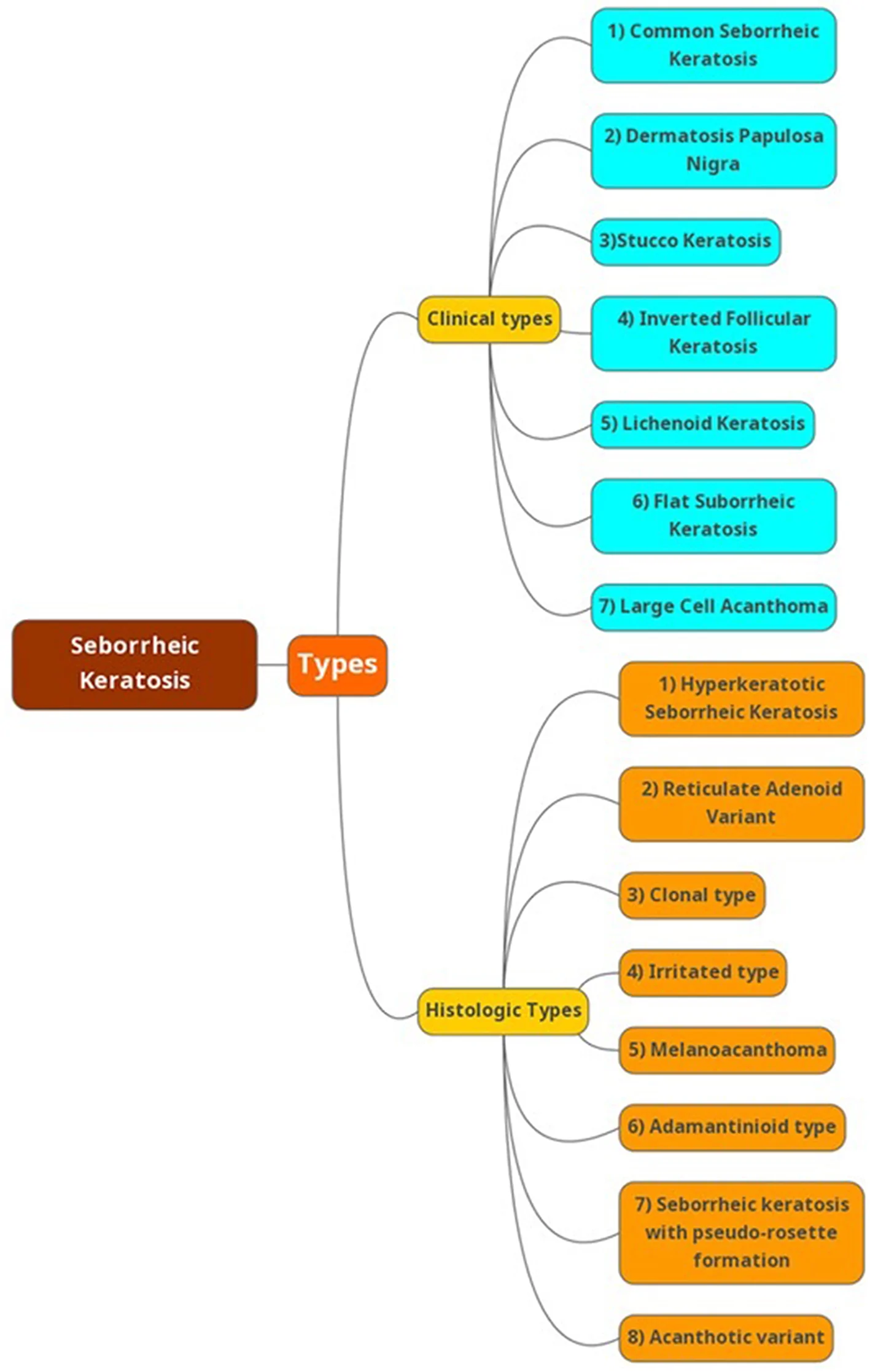
Classification of seborrheic keratosis. Adapted from Khalili et al. (12) under the creative commons attribution license (CC BY 4.0).

This is the most common variant, showing moderate acanthosis, papillomatosis, and hyperkeratosis, predominantly composed of basaloid cells. Numerous pseudo-horn cysts are present, and approximately one-third of cases exhibit pigmentation. A mild lichenoid lymphocytic infiltrate may also be observed (12). The clinical and histopathologic classification of seborrheic keratosis is shown in Figure 4.

### 3.5 Diagnosis

#### 3.5.1 Non-invasive diagnosis

SK lesions that mimic melanocytic neoplasms can be challenging to diagnose with the naked eye. A clinicopathologic review of 8,694 skin biopsies found that 30% of excised SK lesions were misdiagnosed as non-melanocytic skin cancers and 3% as melanoma. Furthermore, 21% of all misdiagnosed cutaneous melanoma cases were initially considered to be SK or benign nevi. An evaluation of 367 BCC cases also revealed that 22% of misdiagnosed cases were, in fact, SK. These findings indicate a tendency toward over diagnosing malignancy, reflecting a cautious approach in skin tumor management. They also highlight the potential for advanced imaging techniques to enhance diagnostic accuracy. In addition, data from SK lesions have played a significant role in the development of artificial intelligence (AI)-based technologies for computerized skin lesion detection (13).

#### 3.5.2 Dermatoscopy

**Figure 5:**
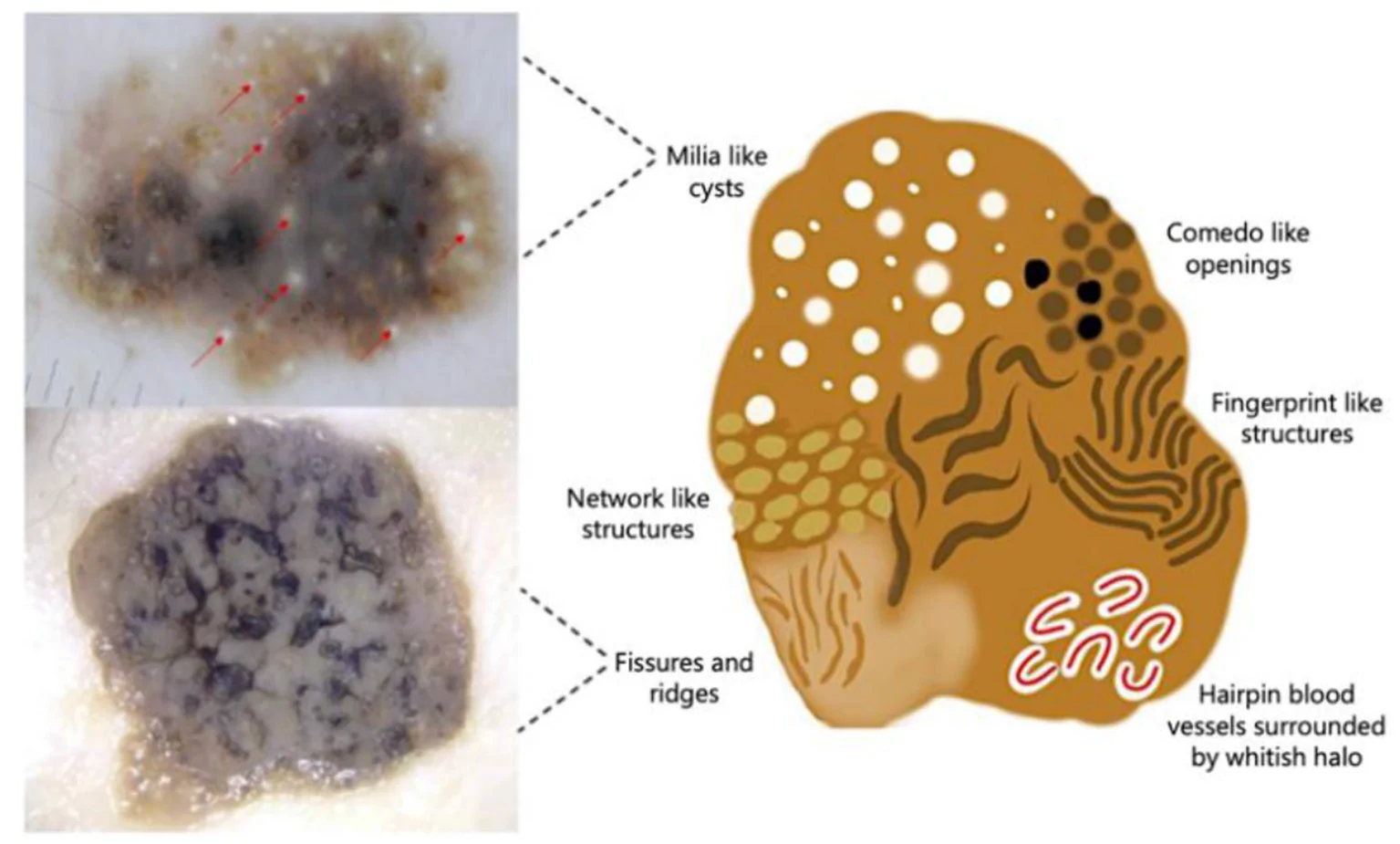
Summary illustration of key dermatoscopy inclusion criteria for seborrheic keratosis (right), along with dermatoscopy images showing milia-like cysts, fissures, and ridge patterns (left). Adapted from Weber et al. (14) under the creative commons attribution license (CC BY 4.0).

Dermatoscopy is a powerful technique that enables real-time visualization of diagnostic features that are not visible to the unaided eye. Although other imaging methods offer higher resolution, dermatoscopy remains the most widely used due to its ease of use and broad availability. Notably, polarized and non-polarized dermatoscopy provide different insights and are best used complementarily. A widely accepted two-step dermatoscopy algorithm for diagnosing SK includes: (1) Exclusion criteria such as pigment network, aggregated globules, and homogeneous blue pigmentation, which suggest a melanocytic lesion. (2) Inclusion criteria including fissures and ridges (cerebriform pattern), comedo-like openings, milia-like cysts, and brown fingerprint-like structures, as illustrated in Figure 5. Further studies have identified additional dermatoscopy features, including hairpin vessels, sharp lesion demarcation, moth-eaten borders, crypts, and exophytic papillary structures. A prospective study of 416 lesions found that the absence of blue-gray/white coloration, the presence of yellowish tones, and mica-like structures improved the identification of SK (14).

In general, the aforementioned features are key diagnostic findings for SK. They are utilized in dermatoscopy assessment methods that have been shown to improve clinical accuracy in distinguishing SK from melanoma that mimics SK. A subset of these features—including hairpin vessels, regularly distributed vascular patterns, and lesions with at least 10% surface area covered by a white halo—can aid in differentiating SK from irritated SCC. However, a consistent global dermatoscopy pattern for atypical SK has not yet been established. A study involving 72 biopsied lesions found that some suspected SK cases actually exhibited non-SK characteristics, such as blue-white veils, polymorphous vessels, granules, and shiny white streaks (14).

Several dermatoscopy-based imaging techniques have been developed to enhance diagnostic precision. These include videodermatoscopy (sequential digital dermatoscopy) and computer-assisted digital dermatoscopy. The latter incorporates an integrated approach to objectively measuring various parameters, which are then analyzed as potential discriminative variables within a diagnostic model. This integration of numerical features, dermatoscopy patterns, and personal metadata was originally applied to melanoma diagnosis, but the methodology is now being extended to SK. When used alongside dermatoscopy data, information obtained from multispectral digital skin lesion analysis devices has also been shown to improve clinical decision-making (14).

#### 3.5.3 Other optical techniques

RCM enables cellular-level visualization of the epidermis and superficial dermis. In RCM analysis, a honeycomb epidermal pattern and densely packed, well-defined dermal papillae at the dermoepidermal junction suggest benign neoplasms. Specific features of SK observed with RCM include epidermal projections, keratin-filled invaginations, cord-like and bulbous projections, corneal pseudocysts, and mixed vascular patterns. However, in recent years, studies have highlighted the challenges in accurately diagnosing SK using RCM due to its clinical variability and limited imaging depth. In a retrospective analysis of 390 patients with clinically suspicious SK, dermatologists using RCM were unable to confirm an SK diagnosis in 25% of cases. Previously, SK had been misdiagnosed as pigmented nevi, and RCM features were reported to be insufficient for differentiating melanoacanthoma from melanoma (15).

OCT also provides subsurface imaging with greater penetration depth than RCM, though at a lower resolution. While its use in managing SK remains limited, reported diagnostic criteria for SK include acanthosis, basal hyperpigmentation, visibility of the dermoepidermal junction, a normal honeycomb pattern, loss of normal epidermal and dermal layers, and widened interpapillary spaces with edged papillae. Given OCT's ability to rapidly assess architectural changes in the epidermis and dermis, it has also been used to monitor clearance of SK after 40% hydrogen peroxide topical solution treatment. In the future, OCT may serve as a valuable alternative or complement to histopathological examination of SK (15).

Several experimental photodynamic approaches have been explored for SK identification. A pilot study found that autofluorescence analysis using a smartphone RGB (red, green, blue) camera under 405 nm LED excitation could differentiate 15 SK lesions from 36 pigmented nevi, BCC, and melanoma lesions with high sensitivity and specificity. Near-infrared autofluorescence revealed that SK lesions emitted significantly brighter signals compared to normal skin. Raman spectroscopy, which assesses structural changes in lipid proteins, has also been used to distinguish melanoma from pigmented nevi, BCC, and SK. A recently developed low-cost handheld Raman device achieved moderate performance in differentiating SK from melanoma. Photodynamic techniques and thermovision studies have demonstrated that SK lesions exhibit a lower average temperature than healthy skin, with a significantly different temperature gradient compared to BCC (15).

#### 3.5.4 Artificial intelligence

**Figure 6:**
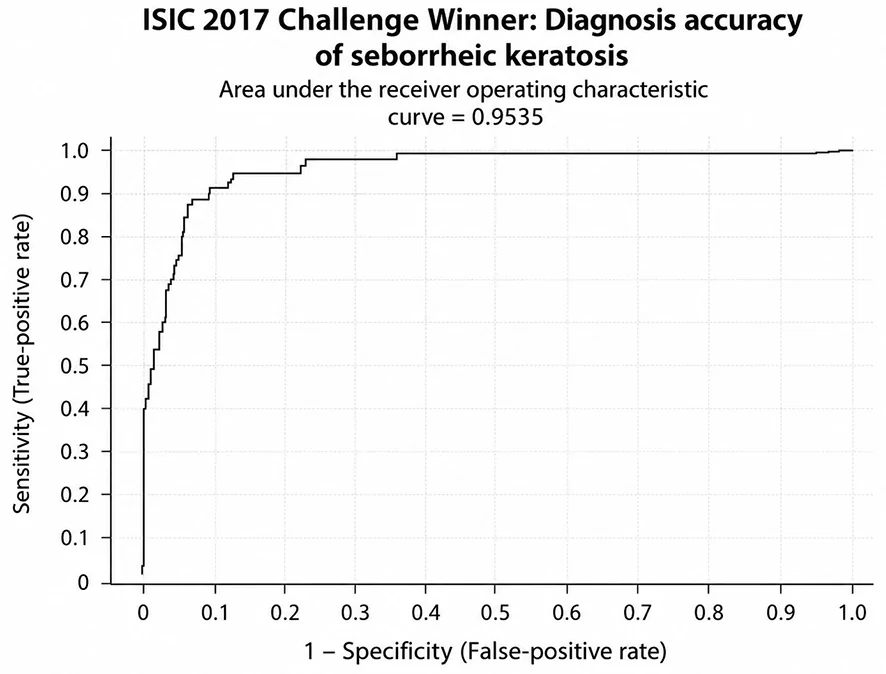
Receiver operating characteristic curve showing the performance of artificial intelligence algorithms for detecting seborrheic keratosis in the 2017 international skin imaging collaboration challenge. Adapted from Marchetti et al. (16) under the creative commons attribution license (CC BY 4.0).

The SK image is often used to train AI algorithms that are capable of distinguishing malignant skin tumors from benign ones. The 2017 International Skin Imaging Collaboration challenge dataset includes 50 melanomas, 50 nevi, and 50 SKs, resulting in a deep learning model with an area under the receiver operating characteristic curve (AUC) of 0.954 for SK (Figure 6) (16).

Machine-based systems and deep learning models that outperform doctor readers often categorize SK as a distinct category and report AUC values ranging from 0.77 to 0.96. Others stratify the results by increasing the risk category and classify SK as a low-risk designation. Several algorithms, based on various deep learning frameworks such as convolutional neural networks and Gabor wavelet-based learning, have been trained on SK vs. BCC or melanoma-only datasets. These computational tools show significant promise in improving clinical SK detection (16).

#### 3.5.5 Confocal laser scan microscopy (CLSM)

Another diagnostic method includes CLSM. This method depicts lesion depth, providing a three-dimensional impression for further diagnostic certainty beyond the two-dimensional dermatoscopy. CLSM does not compete with dermatoscopy but rather complements it. It can reveal skin cellular structures up to a depth of approximately 250–300 μm. This method is highly effective in diagnosing SK. Its drawback is that the area that can be imaged is relatively small, which causes some difficulties, especially for larger structures. Structures detectable by dermatoscopy show correlations with CLSM. Milium-like cysts are associated with keratin pseudocysts in the papillary space, extending into the suprabasal plane. Openings resembling comedones can be seen as superficial epidermal invaginations containing keratin. Not all SK subtypes can be reliably diagnosed with CLSM, particularly due to the limited depth of penetration of this method (17).

### 3.6 Treatment

SK is primarily removed for cosmetic reasons. Common treatment options include cryotherapy, shave excision, electrodesiccation, and curettage. Cryotherapy is the most frequently used method, especially for flat or thin lesions, and is often preferred by patients due to its minimal postoperative care requirements. When the clinical diagnosis is certain, cryotherapy is recommended (18).

Curettage or shave excision may be performed under local anesthesia using infiltrative 1% lidocaine administered intradermally or subcutaneously with a fine needle. In selected superficial lesions, topical anesthetic cream, particularly lidocaine/prilocaine cream applied under occlusion for approximately 1–2 h, may provide adequate dermal anesthesia and reduce the need for injection. Both curettage and shave excision have demonstrated cosmetic outcomes comparable to cryotherapy. Post-procedure care after curettage is usually simple and outpatient-based, consisting of superficial hemostasis, wound cleansing and drying, application of a protective ointment or topical antibiotic when clinically indicated, and short-term dressing care at home until re-epithelialization occurs (18).

Electrodesiccation, either alone or in combination with curettage, also requires anesthesia and is associated with potential side effects such as scarring, post-procedural depigmentation, and recurrence. Various lasers including carbon dioxide (CO_2_), erbium:yttrium aluminum garnet (Er:YAG), 755 nm alexandrite, and 532 nm diode lasers have been used to treat SK with varying success (18).

More recently, 40% hydrogen peroxide topical solution has been approved by the Food and Drug Administration (FDA) as a treatment option, and 30%−50% aqueous nitric-zinc oxide solution with organic acids solution has demonstrated good clinical responses with no recurrence in clinical trials. However, none of these therapies have been thoroughly evaluated in randomized clinical trials. Treatment selection depends on factors such as lesion size and thickness, skin type, and clinical expertise (18).

Other topical agents, including 5% imiquimod cream, 5% calcium dobesilate cream, and 3% diclofenac gel, have shown variable response rates. Ablative lasers such as Er:YAG have produced good outcomes with minimal side effects, although cost may be a limiting factor. CO_2_ lasers are also effective but are associated with higher risks of scarring and post-inflammatory hyperpigmentation. Additional lasers studied include PDL and the 1,064 nm Q-switched Nd:YAG. Nanosecond pulsed electric field technology, also known as NPS, is a non-thermal, drug-free, energy-based treatment that has shown promising results. In one study, physicians rated 82% of treated SKs as clear or mostly clear, and 78% of patients reported being satisfied or mostly satisfied, with no adverse effects reported (18).

In addition, conventional and emerging therapeutic modalities are compared in terms of efficacy, recurrence, adverse effects, and clinical applicability to facilitate evidence-based decision-making in daily practice (Table 2).

**Table 2:** Comparison of conventional and emerging treatments for seborrheic keratosis.

Treatment modality	Type	Advantages	Limitations	Adverse effects	Recurrence data	Evidence strength	Clinical relevance
Cryotherapy	Conventional destructive therapy	Widely available, simple, quick, and effective for thin lesions	Operator-dependent depth control; less ideal for multiple or thicker lesions	Pain, blistering, hypopigmentation, post-inflammatory pigment alteration	Variable; depends on lesion thickness and treatment adequacy	Established	Common first-line office-based treatment
Curettage/shave excision	Conventional procedural therapy	Immediate lesion removal; tissue available for histopathology	Requires anesthesia and wound care	Bleeding, scarring, pigmentary change	Possible if incompletely removed	Established	Useful when diagnosis is uncertain or histology is needed
Electrodesiccation	Conventional destructive therapy	Effective for raised lesions	Requires local anesthesia; cosmetic results may vary	Scarring, depigmentation, irritation	Possible recurrence	Established	Useful but sometimes less favorable cosmetically
CO2 laser	Ablative laser therapy	Effective lesion ablation; useful for selected lesions	Expensive and less accessible	Erythema, scarring, post-inflammatory hyperpigmentation	Limited long-term comparative data	Moderate	Useful for selected cosmetic indications
Er:YAG laser	Ablative laser therapy	Good healing and favorable cosmetic outcomes in some studies	Equipment cost and availability	Erythema, transient irritation	Limited long-term data	Moderate	Potentially preferable in cosmetically sensitive areas
KTP/PDL/Nd:YAG lasers	Non-ablative/selective laser therapy	Useful in selected SK or DPN lesions	Data still limited and heterogeneous	Pain, hypo-/hyperpigmentation, variable response	Incompletely defined	Low to moderate	Promising in selected patients
Hydrogen peroxide 40% w/w topical solution	Topical therapy	Non-surgical office-based treatment	Modest complete clearance rate; limited practical use in some settings	Stinging, erythema, local irritation	Limited long-term data	Moderate	Historically important but currently less used
30–50% aqueous nitric-zinc oxide solution with organic acids	Emerging topical therapy	Favorable cosmetic profile; easy application; encouraging early response	Evidence limited to small studies	Mild discomfort or irritation	Short-term recurrence appears low in early studies	Low	Promising non-invasive option
65% or 80% TCA solution ± formic acid topical solution	Emerging topical chemical therapy	Good response in selected lesions, including some darker skin types	Irritation risk; protocol not standardized	Burning, erythema, post-inflammatory pigmentation	Limited data	Low	Potential alternative when procedures are less desirable
Annona muricata extract	Investigational botanical topical therapy	Non-invasive and potentially well tolerated	Evidence still preliminary and based on limited studies	Safety profile needs stronger validation	Long-term recurrence unknown	Very low	Experimental option requiring further study
Nano-Pulse Stimulation (NPS)	Emerging energy-based therapy	Non-thermal and drug-free; favorable early clearance and satisfaction	Limited comparative evidence and short follow-up	Generally mild local reactions; long-term safety still uncertain	Longer-term recurrence data lacking	Low to moderate	Promising novel modality
5% imiquimod cream/3% diclofenac gel/tazarotene 0.1% cream BID/vitamin D analogs	Other topical therapies	Non-invasive and easy to apply	Inconsistent efficacy across studies	Irritation, erythema, limited response	Poorly defined	Low	Not yet established as standard treatment

Overall, conventional destructive therapies remain the most established treatment options for SK; however, their use may be limited by discomfort, pigmentary alteration, and cosmetic concerns. By contrast, newer modalities such as 30%−50% aqueous nitric-zinc oxide solution with organic acids, 0.3% Annona muricata L. seed extract cream, and NPS appear promising, but the supporting evidence is still limited by small sample size, short follow-up, and the relative lack of randomized comparative studies.

### 3.7 Recent therapies

Due to the benign nature of SK, treatment is generally not necessary. However, treatment may be desired or planned, primarily for cosmetic reasons. As such, the risk of complications and post-treatment cosmetic outcomes should be key considerations when selecting a therapy (17).

Available treatments are mostly limited to destructive therapies and minor surgical techniques, including cryotherapy, shave excision, and electrodesiccation with curettage. Many of these methods are poorly tolerated by patients with multiple lesions and are more likely to cause post-inflammatory pigmentation in darker skin types. ESKATA^®^, a 40% topical hydrogen peroxide topical solution and the first FDA-approved treatment for SK, was discontinued commercially in the U.S. in 2019. This highlights the need for more convenient and cosmetically acceptable SK therapies (19).

### 3.8 Local destructive methods

Large, highly pigmented lesions located in exposed areas can cause significant cosmetic concerns and may be removed if desired. Patients with numerous SK lesions often opt for removal. The current approach involves curettage using a sharp or ring curette. Patients should be informed about the potential for scarring and recurrence if the lesion is not completely removed, and that lesions may recur repeatedly. Alternatively, lesions can be removed by shave excision with a scalpel, though recurrence is also possible in such cases. Treatment is less commonly indicated for medical reasons. In cases of persistent mechanical irritation, for example due to clothing in areas like the waistband or bra line, lesions should be removed as irritation can lead to recurrent inflammation, bleeding, and increased pruritus. If SK interferes with function—such as when located on the eyelids or ears—treatment is recommended. If the diagnosis is uncertain, the lesion should be excised and submitted for histopathological examination to rule out malignancy (17).

### 3.9 Topical treatments

Due to the high prevalence of SK and strong patient interest in non-invasive therapies, various attempts have been made to treat these lesions topically. However, no topical treatment has yet proven to be consistently reliable and widely applicable. There have been case reports of successful treatment with 3% diclofenac gel, but other studies have concluded that once-daily applications of tazarotene 0.1% cream BID, calcipotriol, and 5% imiquimod cream are not promising options. Notably, using tazarotene 0.1% cream BID showed visible improvement in 7 out of 15 subjects, although initial irritation and erythema occurred. Vitamin D3 ointment applied once or twice daily for more than three months resulted in remission in only 30.2% of 116 subjects. However, other newly investigated agents have shown better efficacy. Among the most notable is a 40% hydrogen peroxide topical solution, studied in two large placebo-controlled clinical trials involving nearly 1,000 patients and marketed in the U.S. as Eskata^®^. Two applications spaced three weeks apart led to clearance of at least three out of four treated SKs in approximately 30% of patients. Other topical combinations, such as alpha- and beta-hydroxy acids, urea, and thuja demonstrated a 99% success rate in a study with 20 subjects. Topical calcium dobesilate 5% cream and combinations of 65 or 80%TCA solution and formic acid topical solution also proved effective, though larger clinical trials are still lacking. Therefore, these treatments are not yet considered established therapies for SK (17).

In vitro studies by Neel et al. showed that explanted SK tissue completely dissolved after incubation with an AKT inhibitor (A-443654). Another AKT inhibitor, SM-020, is currently under investigation for topical use in treating SK (NCT05136144), potentially offering a promising option due to the AKT signaling pathway's relevance to SK development. Early studies on topical therapies have shown promising results. For instance, 30–50% aqueous nitric-zinc oxide solution with organic acids demonstrated significant lesion clearance with minimal discomfort and no recurrence. A new TCA and formic acid topical solution also showed complete response in 90% of patients, though it was associated with more drug-related side effects. Focal TCA peeling also yielded good clinical responses in patients with darker skin types (Fitzpatrick IV–V). By contrast, tazarotene 0.1% cream BID showed improvement in less than half of the patients, while 12% ammonium lactate lotion (Lac-Hydrin), applied twice daily only slightly improved lesions. Topical clearance has also been reported with calcium dobesilate, 5% imiquimod cream, and 3% diclofenac gel. However, a recent meta-analysis found no solid evidence supporting the efficacy of topical vitamin D analogs (20).

Another emerging non-invasive approach is the use of 0.3% Annona muricata L. seed extract cream, which may be effective in treating SK. Soursop seed extract contains acetogenins, compounds known for their selective cytotoxicity against abnormal cells. This property may help reduce the size and number of lesions without irritating surrounding healthy skin. Topical application is convenient and avoids the side effects often associated with invasive procedures like cryotherapy or curettage. A 2023 study by Yesie Elvira showed that a 0.3% soursop seed extract cream led to significant clinical improvement in patients with SK. The proposed mechanism involves promoting skin regeneration and exfoliation of excess keratin. This cream thus has the potential to become a safe and effective alternative therapy for patients with SK (21).

### 3.10 Laser and light-based therapy

Ablative laser therapy, though relatively expensive, has been used to treat SK. In one observational study, CO_2_ laser treatment was applied to lesions unresponsive to a combination cream containing 4% hydroquinone, showing an average efficacy of 84% among 53 patients. In some cases of DPN, CO_2_ laser successfully removed SK lesions without scarring or significant discomfort. Additionally, two cases of irritated SK treated with CO_2_ laser showed no recurrence between 6 and 12 months follow-up. A comparative study between erbium:YAG (Er:YAG) laser and cryotherapy in 42 patients reported better healing and less post-inflammatory hyperpigmentation with Er:YAG, though with more erythema (22).

Non-ablative lasers have also shown promise. Split-face randomized trials comparing potassium titanyl phosphate (KTP) laser with electrodesiccation in DPN patients found KTP to be effective and better tolerated, although post-treatment hypo- and hyperpigmentation were common. A prospective pilot study using pulsed dye laser (PDL) showed comparable results to curettage and electrodesiccation, but PDL was the most painful and did not reduce post-treatment pigmentation. Nd:YAG (1,064 nm) laser was successful in two DPN cases without side effects. Other light-based therapies include fractional photothermolysis, intense pulsed light (IPL), Q-switched, and picosecond lasers. Recently, aminolevulinate photodynamic therapy (ALA-PDT) has also been effective in treating giant SK (23).

### 3.11 Oral therapy

Oral 1,25-dihydroxyvitamin D3 has been tested as a non-topical treatment. In a case study involving 51 patients, those receiving a high dose (0.5 μg/day) showed rapid transformation of lesions from dark brown papules to pigmented macules or atrophic scars. Patients on a lower dose (0.25 μg/day) showed significant fading and shrinking of lesions without erythema. Although promising for small SK lesions, these results require validation in larger studies to confirm efficacy (17).

## 4 Conclusion

SK remains the most prevalent benign epidermal tumor with significant clinical and cosmetic implications. Recent advances in molecular research, particularly involving the AKT signaling pathway and APP, have provided new insights into its pathogenesis. Diagnostic innovations, including dermatoscopy, RCM, OCT, and AI, have markedly improved the accuracy of differentiating SK from malignant lesions. In terms of therapy, while traditional destructive methods such as cryotherapy, curettage, excision, and laser remain effective, they are often associated with discomfort and post-treatment pigmentation changes. Emerging non-invasive modalities, such as 30%−50% aqueous nitric-zinc oxide solution with organic acids, 65 or 80% TCA solution/chemical peel, 0.3% Annona muricata L. seed extract cream, and NPS technology offer promising alternatives with favorable cosmetic outcomes and minimal side effects. Nonetheless, larger randomized controlled trials are needed to validate long-term efficacy, optimize treatment algorithms, and broaden accessibility. The integration of molecular understanding, advanced diagnostics, and innovative therapies underscores the evolving landscape of SK management, with growing emphasis on patient-centered, safe, and cosmetically acceptable solutions.

## References

[1] Moreno-Ramírez D Raya-Maldonado J Morales-Conde M Ojeda-Vila T Martín-Gutiérrez FJ Ruíz-de-Casas A . Increasing frequency of seborrheic keratosis diagnoses as a favorable consequence of teledermatology-based skin cancer screening: a cross-sectional study of 34,553 patients. Am J Clin Dermatol. (2017) 18:681–5. doi: 10.1007/s40257-017-0283-z 28397109

[2] Sun MD Halpern AC. Advances in the etiology, detection, and clinical management of seborrheic keratoses. Dermatology. (2022) 238:205−17. doi: 10.1159/000517070 34311463

[3] Kao S Kiss A Efimova T Friedman AJ. Managing seborrheic keratosis: evolving strategies and optimal therapeutic outcomes. J Drugs Dermatol. (2018) 17:933–40. 30235378

[4] Bianchi MG Santos A Cordioli E. Benefits of teledermatology for geriatric patients: population-based cross-sectional study. J Med Internet Res. (2020) 22:e16700. doi: 10.2196/16700 32314966 PMC7201316

[5] Reddy R Davidova L Bhattacharyya I Cohen DM Islam MN Fitzpatrick SG. Dermatologic lesions submitted to an oral and maxillofacial pathology biopsy service: an analysis of 2487 cases. Head Neck Pathol. (2018) 12:493–9. doi: 10.1007/s12105-018-0885-7 29340950 PMC6232221

[6] Hirobe S Otsuka R Iioka H Quan YS. Kamiyama F, Asada H, et al. Clinical study of a retinoic acid-loaded microneedle patch for seborrheic keratosis or senile lentigo. Life Sci. (2017) 168:24–7. doi: 10.1016/j.lfs.2015.12.051 26757104

[7] Komalasari DN Djawad K Nurdin AR Paturusi II. Unusual clinical manifestation of seborrheic keratosis on the scalp successfully treated with topical trichloroacetic acid: an atypical case report. Pan Afr Med J. (2020) 28:98. doi: 10.11604/pamj.2020.37.98.26090 33425131 PMC7757303

[8] Hafner C Landthaler M Mentzel T Vogt T. FGFR3 and PIK3CA mutations in stucco keratosis and dermatosis papulosa nigra. Br J Dermatol. (2010) 162:508–12. doi: 10.1111/j.1365-2133.2009.09488.x 19845664

[9] Battistella M Peltre B Cribier B. Composite tumors associating trichoblastoma and benign epidermal/follicular neoplasm: another proof of the follicular nature of inverted follicular keratosis. J Cutan Pathol. (2010) 37:1057–63. doi: 10.1111/j.1600-0560.2009.01341.x 19615018

[10] Yoon JH Baek EJ Park EJ Kim KH. Comparative study of treatment methods for benign lichenoid keratosis of the face. Dermatol Ther. (2022) 5:e15419. doi: 10.1111/dth.15419 35246904

[11] Park JB Jung JH Kwon DI Seong SH Suh KS Jang MS. 25036 Dermoscopic features of large cell acanthoma. J Am Acad Dermatol. (2021) 85:AB52. doi: 10.1016/j.jaad.2021.06.232

[12] Khalili M Meymandi SS Amiri R Aflatoonian M Alimortazavi S. Clinicopathological evaluation of seborrheic keratosis lesions in patients referred to Afzalipour hospital, Kerman, Iran. J Skin Stem Cell. (2022) 9:e122310. doi: 10.5812/jssc.122310

[13] Minagawa A. Dermatoscopy-pathology relationship in seborrheic keratosis. J Dermatol. (2017) 44:518–24. doi: 10.1111/1346-8138.13657 28447350

[14] Weber P Tschandl P Sinz C Kittler H. Dermatoscopy of neoplastic skin lesions: recent advances, updates, and revisions. Curr Treat Options Oncol. (2018) 19:56. doi: 10.1007/s11864-018-0573-6 30238167 PMC6153581

[15] Guo A Chen J Yang C Ding Y Zeng Q Tan L. The challenge of diagnosing seborrheic keratosis by reflectance confocal microscopy. Skin Res Technol. (2018) 24:663–6. doi: 10.1111/srt.12582 29797357

[16] Marchetti MA Liopyris K Dusza SW Codella NCF Gutman DA Helba B . Computer algorithms show potential for improving dermatologists' accuracy to diagnose cutaneous melanoma: results of the international skin imaging collaboration 2017. J Am Acad Dermatol. (2020) 82:622– 7. doi: 10.1016/j.jaad.2019.07.016 31306724 PMC7006718

[17] Barthelmann S Butsch F Lang BM Stege H Großmann B Schepler H . Seborrheic keratosis. J Dtsch Dermatol Ges. (2023) 21:265–77. doi: 10.1111/ddg.14984 36892019

[18] Zaresharifi S Robati RM Dadkhahfar S Forouzanfar MM Zaresharifi N. Efficacy and safety of cryotherapy, electrodesiccation, CO2 laser, and Er:YAG laser in the treatment of seborrheic keratosis. Dermatol Ther. (2021) 34:1–7. doi: 10.1111/dth.15083 34342933

[19] Keaney T. Eskata (40% hydrogen peroxide solution) for the treatment of seborrheic keratoses. J Drugs Dermatol. (2019) 18:s172. 31336412

[20] Takekawa C Fukumoto T Haraoka G. Terashi H. Combination treatment algorithm for pigmentary disorders of the face: a prospective observational study in Asian patients. J Plast Reconstr Aesthet Surg. (2021) 74:370–76. doi: 10.1016/j.bjps.2020.08.131 33046430

[21] Elvira Y. Pengaruh Pemberian Krim Ekstrak Biji Sirsak (Annona muricata L.) 0.3% terhadap Keratosis Seboroik (thesis). Faculty of Medicine, Universitas Sumatera Utara, Medan (2023).

[22] Tran M Richer V. Elective treatment of dermatosis papulosa nigra: a review of treatment modalities. Skin Therapy Lett. (2020) 25:1–5. 33017107

[23] Zhang H Wei G Zhang C Xu Q Zhang C. Aminolevulinate photodynamic therapy (ALA-PDT) for giant seborrheic keratosis of the head: a case report. Photodiagnosis Photodyn Ther. (2020) 32:102015. doi: 10.1016/j.pdpdt.2020.102015 32950725

[24] Lin TL Lee KH Karmakar R Mukundan A Sundarraj J Lu CT . Artificial intelligence-assisted dermatologic screening: epidemiology and clinical features of basal cell carcinoma, squamous cell carcinoma, seborrheic keratosis and actinic keratosis. Bioengineering. (2025) 12:1258. doi: 10.3390/bioengineering12111258 41301214 PMC12650624

[25] Lin TL Mukundan A Karmakar R Avala P Chang WY Wang HC. Hyperspectral imaging for enhanced skin cancer classification using machine learning. Bioengineering. (2025) 12:755. doi: 10.3390/bioengineering12070755 40722447 PMC12292285

[26] Jung JM Noh TK Jo SY Kim SY Song Y Kim YH . Guanine deaminase in human epidermal keratinocytes contributes to skin pigmentation. Molecules. (2020) 25. doi: 10.3390/molecules25112637 32517074 PMC7321356

[27] Cheong KA Lee AY. Guanine deaminase stimulates ultraviolet-induced keratinocyte senescence in seborrhoeic keratosis via guanine metabolites. Acta Derm Venereol. (2020) 100:adv00109. doi: 10.2340/00015555-3473 32215662 PMC9128948

